# Convergent evolution in the Euarchontoglires

**DOI:** 10.1098/rsbl.2018.0366

**Published:** 2018-08-01

**Authors:** Philip J. R. Morris, Samuel N. F. Cobb, Philip G. Cox

**Affiliations:** 1 Hull York Medical School, University of Hull, Hull HU6 7RX, UK; 2 Department of Archaeology and Hull York Medical School, University of York, York YO10 5DD, UK

**Keywords:** convergent evolution, cranium, mandible, morphology, aye-aye, rodents

## Abstract

Convergence—the independent evolution of similar phenotypes in distantly related clades—is a widespread and much-studied phenomenon. An often-cited, but hitherto untested, case of morphological convergence is that between the aye-aye and squirrels. The aye-aye (*Daubentonia madagascariensis*) is a highly unusual lemuriform primate that has evolved a dentition similar to that of rodents: it possesses large, ever-growing incisors which it uses to strip the bark from trees in order to feed on wood-boring beetle larvae. Indeed, such is the similarity that some of the earliest classifications of the aye-aye placed it in the squirrel genus *Sciurus*. Here, we aimed to test the degree of convergence between the skulls and lower jaws of squirrels and the aye-aye. Three-dimensional landmarks were recorded from the crania and mandibles of 46 taxa representing the majority of families in the Euarchontoglires. Results were plotted as phylomorphospaces and convergence measures were calculated. The convergence between squirrels and the aye-aye was shown to be statistically significant for both the cranium and mandible, although the mandibles seem to converge more closely in shape. The convergence may indicate strong functional drivers of morphology in these taxa, i.e. the use of the incisors to produce high bite forces during feeding. Overall, we have shown that this classic case of convergence stands up to quantitative analysis.

## 1. Introduction

Convergence, the independent evolution of similar phenotypes in phylogenetically distinct lineages, is an important and widespread evolutionary process [1,2], and one that has been recognized since the beginnings of evolutionary biology as a field [3]. Convergent evolution is often thought to represent adaptation of distantly related organisms to a similar environment, but may also indicate the presence of a biological constraint limiting the available range of phenotypes [4]. Recent developments in the quantification of convergence [2,5] have enabled researchers not only to identify instances of convergent evolution but also to test its statistical significance (e.g. [6–9]). Therefore, iconic examples of convergence, hitherto classified as such qualitatively, can now be tested quantitatively.

One such classic example of convergence is that of the aye-aye and rodents. The aye-aye (*Daubentonia madagascariensis*) is a lemuriform primate, native to Madagascar. Its unusual diet, which includes wood-boring beetle larvae [10], has driven a number of morphological adaptations, such as acute hearing and an elongate middle digit for percussive foraging, and enlarged, ever-growing incisors for stripping the bark from trees to reveal larval burrows [11]. In fact, the entire dentition, not just the incisors, is strikingly rodent-like, with the dental formula being 1.0.1.3 in the upper jaw and 1.0.0.3 in the lower [12]. Indeed, so close is the resemblance to rodents, that in some of the earliest taxonomies of mammals, the aye-aye was classified as a squirrel, and placed in the genus *Sciurus* (e.g. [13,14]).

Although the morphological similarities between the aye-aye and sciurid rodents have been noted by many authors [15,16], the degree of convergence between them has never been formally tested. In this study, we used geometric morphometric methods (GMM) to test the *a priori* hypothesis that both the cranium and the mandible of the aye-aye are convergent with those of squirrels. Although it is possible to identify convergence without an *a priori* hypothesis using multivariate data, such methods are not suitable for the high-dimensional shape data gathered here [9]. Morphological similarity between squirrels and the aye-aye, despite their phylogenetic separation, is predicted based on the previous misclassification of the aye-aye as a squirrel, and also because both groups engage in mechanically demanding feeding activities with their anterior teeth [17]. We predicted that the bony elements of the skull, not just the teeth, would show morphological convergence owing to the structural constraints of housing enormously enlarged incisors and the functional constraints of using the incisors to generate high bite forces.

## 2. Material and methods

MicroCT scans of the crania and mandibles of 46 species of Euarchontoglires were obtained, either from the online repository Morphosource (www.morphosource.org), or by imaging osteological specimens from museum collections. Virtually reconstructed surfaces of each specimen were created with the segmentation editor of Avizo 8.0 (FEI, Hillsboro, OR, USA), and 22 cranial and 16 mandibular three-dimensional landmarks were collected from the left side of each surface. GMM analyses were implemented in MorphoJ [18]. Further details of sample choice, landmarking methods and GMM are given in the electronic supplementary material, methods. Specimens, landmark coordinates and PC scores are listed in electronic supplementary material, datafile S1.

**Figure 1.**
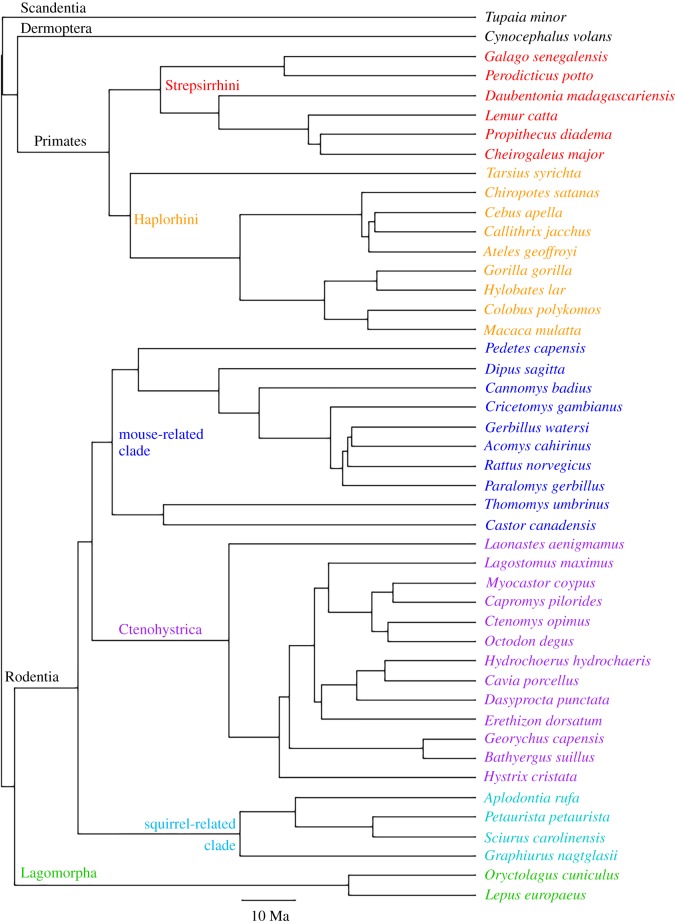
Phylogeny of Euarchontoglires taxa used in this analysis. Topology and dating compiled from Bininda-Emonds *et al.* [19], Arnold *et al.* [20] and Fabre *et al.* [21] Scale bar = 10 million years. Colour coding of taxa matches figure 2.

A phylogeny of the sample species (figure 1) was constructed from previously published data [19–21], and was combined with the morphometric data to construct a phylomorphospace, using the *phytools* package (v. 0.6–44) in R (v. 3.4.2) [22,23]. The degree of convergence between the crania and the mandibles of the aye-aye and the two squirrels in the sample was determined using Stayton's convergence measure *C*_1_ [2]. The significance of the convergence was assessed by comparing the metrics to values obtained from 1000 simulations of evolution under a Brownian motion model. Convergence tests were conducted using the *convevol* R package (v. 1.1) [2].

## 3. Results

**Figure 2.**
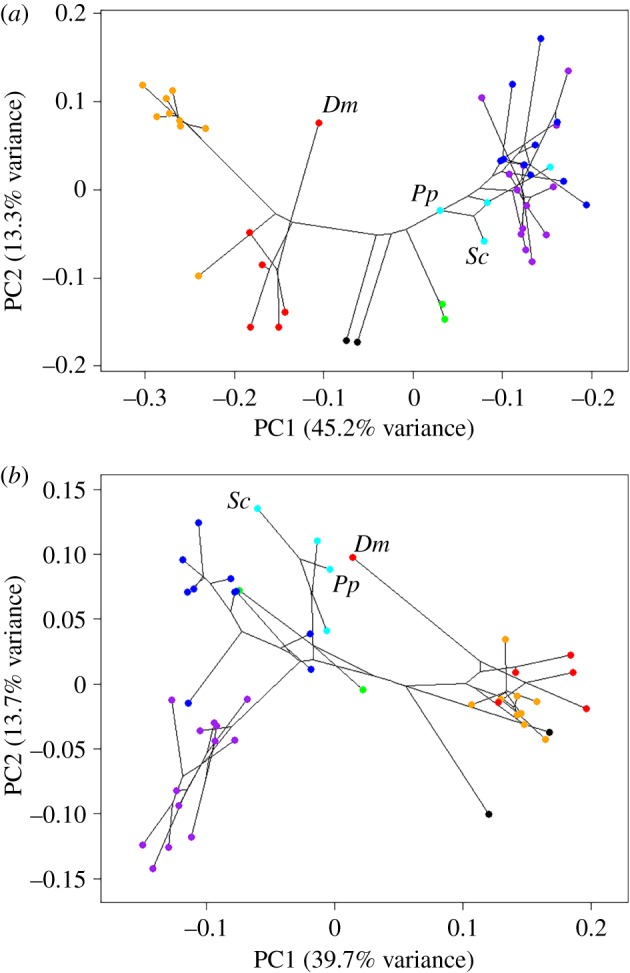
Phylomorphospace showing first two principal components of variation of (*a*) cranial and (*b*) mandibular morphology in Euarchontoglires. Key: red, strepsirrhine primates; orange, haplorhine primates; black, treeshrew and colugo; green, lagomorphs; cyan, squirrel-related rodents; blue, mouse-related rodents; purple, ctenohystrican rodents. *Dm*, *Daubentonia madagascariensis*; *Pp*, *Petaurista petaurista*; *Sc*, *Sciurus carolinensis*.

The first principal component in the cranial analysis (figure 2*a*) shows a clear split between Glires and primates, with the treeshrew and colugo positioned between them. This axis represents a shift from a skull with an elongated rostrum and a flattened cranial vault (positive values, rodents) to a more rounded and taller skull with a flatter face (negative values, primates). Along the second principal component, taxa at the negative extreme of the axis (lagomorphs, prosimians) tend to have flexed cranial bases and relatively large orbits, while taxa at the positive extreme (anthropoid primates, hard-object feeding rodents) have flatter skulls with comparatively smaller orbits. The aye-aye is notably separated from its closest relatives, the strepsirrhines, and is found almost midway between the primates and rodents on PC1, and towards the positive end of PC2. Significant convergence was calculated between the aye-aye and the two sciurid taxa, with a *C*_1_ value of 0.394 (*p* < 0.001), indicating that evolution has closed the distance between the aye-aye and squirrel lineages by almost 40%.

The first principal component of the mandibular analysis (figure 2*b*) again shows a clear distinction between Euarchonta and Glires. The primates, treeshrew and colugo are found towards the positive end of PC1 and are distinguished by a tall coronoid process but only a small angular process, whereas the rodents and lagomorphs at the other end of the axis have a much more prominent angle but a lower coronoid process. The aye-aye is located amongst the rodents rather than the primates, and is particularly close to the squirrel-related rodents on both PC1 and PC2. Significant convergence between the mandibles of the aye-aye and the squirrels was found (*C*_1_ = 0.223; *p* < 0.01), with an average of 22% convergence between their respective lineages. Shape changes along PC axes are shown in electronic supplementary material, figure S2.

## 4. Discussion

The results of this study show that both the cranium and the mandible of the aye-aye are morphologically convergent with those of sciurid rodents, supporting the *a priori* hypothesis of this study (see electronic supplementary material, figure S3 for a comparison of aye-aye and squirrel skulls). The *C*_1_ values [2] calculated for the crania and mandibles are statistically significant, indicating that the aye-aye and squirrels are positioned more closely in morphospace than would be expected under a Brownian motion model of evolution. Morphological similarities are not only restricted to the possession of large, ever-growing incisors but also extend to the bony anatomy of the skull (e.g. rostral length and braincase morphology) and lower jaw (e.g. relative positions of the coronoid and condylar processes). Potentially, such convergence may have been driven by the biomechanical demands of incisor gnawing, which squirrels and the aye-aye both use extensively when feeding. The incisors are used by squirrels to penetrate hard nuts [24], and by the aye-aye for stripping tree bark [11]. Thus the aye-aye and squirrels may have converged on a similar morphology to enable efficient operation of the jaws by the masticatory muscles.

The *C*_1_ values suggest that the crania of the aye-aye and squirrels are more convergent than are the mandibles. However, these values refer to the degree of convergence, not the absolute amount of phenotypic evolution that has occurred [2], nor the level of morphological similarity. From inspection of the morphospaces in figure 2, it appears that the aye-aye mandible more closely resembles that of squirrels, than does the cranium. This was expected as the function of the mandible is almost exclusively related to feeding, whereas the skull must perform other functions such as housing the brain and sensory organs. Furthermore, the shape of the mandible has been shown to correlate closely with diet in squirrels [25], especially amongst hard nut specialists [9]. Overall, we have shown that the classic example of convergence between the aye-aye and squirrels stands up to quantitative analysis, at least with regard to the skull and lower jaw. This may go some way to explaining the erroneous classification of the aye-aye in the genus *Sciurus* in some of the first descriptions of this unusual primate [13,14].

The structure of a morphospace is driven by the taxa included within it. Primates and rodents are both highly speciose orders [26] and it was not possible to include all species, or even all genera, in this analysis. Nevertheless, the specimens chosen represent almost all families of Euarchontoglires and, we feel, reflect the predominant cranial and mandibular morphology seen in each family. As such the sample covers the majority of morphological variation found in Euarchontoglires. Given the distinct split between primates and rodents in both the cranial and mandibular analyses, and the clear deviation of the aye-aye from this pattern, we feel that addition of further specimens would only strengthen our conclusions.

## Supplementary Material

Supplementary datafile

## Supplementary Material

Supplementary methods and results

## Supplementary Material

Figure S3

## Supplementary Material

Phylogeny

## Data accessibility

All morphometric data are available in electronic supplementary material, datafile S1. Surface reconstructions or original microCT scans of all specimens are available from www.morphosource.org (doi numbers given in electronic supplementary material, datafile S1).
