# Supplementary material for: Convergent evolution in the Euarchontoglires
Source: Biol Lett. 2018 Aug 1;14(8):20180366. doi: 10.1098/rsbl.2018.0366 (PMC6127122; doi:10.1098/rsbl.2018.0366)
Supplement: Supplementary methods and results [file rsbl20180366supp2.pdf]

Electronic Supplementary Material

Convergent Evolution in the Euarchontoglires

Philip J.R. Morris[1], Samuel N.F. Cobb[2], Philip G. Cox[2]

[1]Hull York Medical School, University of Hull, Hull, HU6 7RX

[2]Hull York Medical School and Department of Archaeology, University of York, York, YO10 5DD

Methods

The sample used in this analysis was chosen from across the phylogeny of Euarchontoglires. With 3,194 species and 616 genera [1], it was not possible to sample this superorder at the specific or generic level, but the specimens were chosen to cover most extant families. In general, one species per family was included, except if the family was particularly speciose (Muridae, Sciuridae, Cercopithecidae) or where the authors already possessed image data for more than one species in a family (Bathyergidae, Caviidae, Leporidae). Species were chosen to represent the predominant cranial and mandibular morphology of the family and uniquely specialised taxa were avoided. In total, the sample comprised 27 rodents, two lagomorphs, one treeshrew, one colugo, and 15 primates, with each species represented by a single specimen. As such, we feel the sample represents the majority of cranial and mandibular morphological variation seen in Euarchontoglires, from the elongate skulls of *Cricetomys gambianus* and *Laonastes aenigmamus* to the flattened faces of *Hylobates lar* and *Gorilla gorilla*.

Approximately half the sample was sourced as osteological specimens from museum collections and then microCT scanned on the X-Tek HMX 160 scanner at the University of Hull, or, in the case of the skull and lower jaw of *Hydrochoerus hydrochaeris* which were too big for microCT, imaged on the medical CT scanner at The York Hospital. The resulting images (or surface reconstructions derived from them, depending on institutional permission) were uploaded to www.morphosource.org and have been made freely available for download [2]. The remaining specimens in the sample were downloaded directly from www.morphosource.org as stacked microCT slices [3-6]. Virtual 3D surfaces were reconstructed from each stack of image data using Avizo 8.0 (FEI, Hillsboro, OR, USA).

22 cranial and 16 mandibular three-dimensional landmarks were collected from the left side of each surface (Table S1 and Figure S1 below). Landmarks were collected from only one side in order to reduce error that might be introduced by attempting to reconstruct an appropriate position of the hemi-mandibles in taxa with an unfused symphysis. All sets of landmarks were superimposed and projected to the tangent space by orthogonal projection using a full Procrustes fit, in order to remove the effects of size, rotation and translation between specimens. Principal component analysis (PCA) was used to explore the distribution of the taxa in morphospace.

Convergence tests were undertaken in R using the *convevol* package (Stayton, 2015) using the code in the 'Convergence code' sheet of ESM Datafile S1, the phylogeny in the file 'Euarchglir-tree.txt', the data in the 'Cranial-PC-scores' and 'Mandibular-PC-scores' sheets of ESM Datafile S1.

References

1. Burgin CJ, Colella JP, Kahn PL, Upham NS. 2018 How many species of mammals are there? *J. Mammal.* **99,** 1-14.
2. Cox PG. 2018 Diprotodont mammals, P504. *Morphosource.org.* https://www.morphosource.org/MyProjects/Project/form/project_id/504 (last accessed 17 June 2018).
3. Allen KL. 2013 Allen primate skull collection, P77. *Morphosource.org.* https://www.morphosource.org/Detail/ProjectDetail/Show/project_id/77 (last accessed 17 June 2018).
4. Boyer D. 2017 FMNH specimens, P314. *Morphosource.org.* https://www.morphosource.org/Detail/ProjectDetail/Show/project_id/314 (last accessed 17 June 2018).
5. Gonzalez LA. 2013 Gonzalez skull project, P195. *Morphosource.org.* https://www.morphosource.org/Detail/ProjectDetail/Show/project_id/195 (last accessed 17 June 2018).
6. Lucas L, Copes L. 2013 Lucas & Copes MCZ scans, P116. *Morphosource.org.* https://www.morphosource.org/Detail/ProjectDetail/Show/project_id/116 (last accessed 17 June 2018).
7. Stayton CT. 2015 The definition, recognition, and interpretation of convergent evolution, and two new measures for quantifying and assessing the significance of convergence. *Evolution* **69,** 2140-2153.

Table S1. Descriptions of landmarks used in geometric morphometric analyses.

	#	**Landmark description**
**Cranium**	1	Anteriormost point on internasal suture
	2	Anteriormost point on naso-premaxillary suture
	3	Dorsalmost point on the buccal alveolar margin of the upper incisor
	4	Midpoint of lingual alveolar margin of incisor
	5	Midpoint between anterior margins of incisive foramina
	6	Superiormost border of infraorbital foramen
	7	Posteriormost point on internasal suture
	8	Point on frontal suture where it crosses the anterior orbital margin
	9	Dorsalmost point on orbital margin.
	10	Posteriormost margin of the dorsal orbit on the frontal bone
	11	Antero-dorsal point on margin of optic foramen
	12	Anteriormost point of temporal fossa on the jugal arch
	13	Point on ventral margin of zygomatic arch directly inferior to landmark 12
	14	Anteriormost point of infra-temporal fossa on the zygomatic arch
	15	Anteriormost point on the alveolar margin of the mesialmost cheek tooth
	16	Midpoint between anteriormost points of first cheek teeth
	17	Posteriormost point on the alveolar margin of the distalmost cheek tooth
	18	Posteriormost midline point on palatine
	19	Midsagittal point on fronto-parietal suture
	20	Posteriormost point of infra-temporal fossa on the zygomatic arch
	21	Posteriormost point on the dorsal midline of skull
	22	Midpoint of inferior margin of foramen magnum (basion)
**Mandible**	23	Anteriormost point of the dorsal symphyseal margin
	24	Superiormost point on lingual alveolar margin of lower incisor
	25	Inferiormost point on the buccal alveolar margin of the lower incisor
	26	Anteriormost point of the ventral symphyseal margin
	27	Posteriormost point of the dorsal symphyseal margin
	28	Posteriormost point of the ventral symphyseal margin
	29	Anteriormost point on the alveolar margin of the mesialmost cheek tooth
	30	Anteriomost point on the masseteric ridge
	31	Intersection of molar alveolar margin with mandibular ramus
	32	Posteriormost point on the alveolar margin of the distalmost cheek tooth
	33	Tip of coronoid process
	34	Inferiormost point on mandibular notch
	35	Anteriormost point on condyloid process
	36	Posteriormost point on condyloid process
	37	Anteriormost point on curve between condyloid and angular processes
	38	Posteriormost point on angular process

Figure S1. Landmarks used in geometric morphometric analyses shown on surface reconstructions of the skull of *Castor canadensis*. Cranium shown in (*a*) dorsal, (*b*) left lateral and (*c*) ventral views. Left hemimandible shown in (*d*) lateral, (*e*) dorsal and (*f*) medial views. Numbers correspond to landmark descriptions in Table S1.

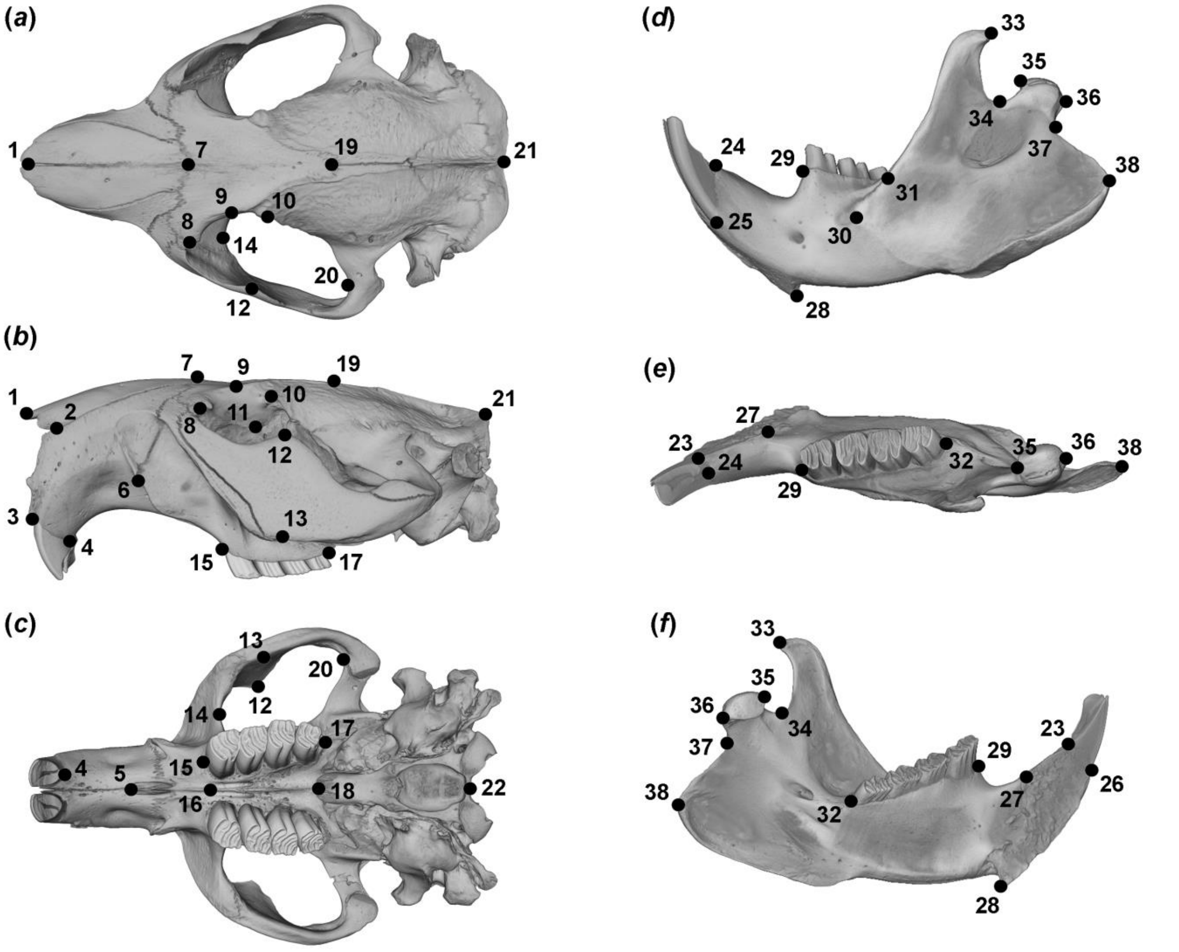

Figure S2. Phylomorphospace showing first two principal components of variation of (*a*) cranial and (*b*) mandibular morphology in Euarchontoglires. Morphological variation along each principal component represented by a surface rendering of the cranium and mandible of *Petaurista petaurista* warped to the positive and negative end of each axis. Key: red, strepsirrhine primates; orange, haplorhine primates; black, treeshrew and colugo; green, lagomorphs; cyan, squirrel-related rodents; blue, mouse-related rodents; purple, ctenohystrican rodents. *Dm*, *Daubentonia madagascariensis*; *Pp*, *Petaurista petaurista*; *Sc*, *Sciurus carolinensis*.

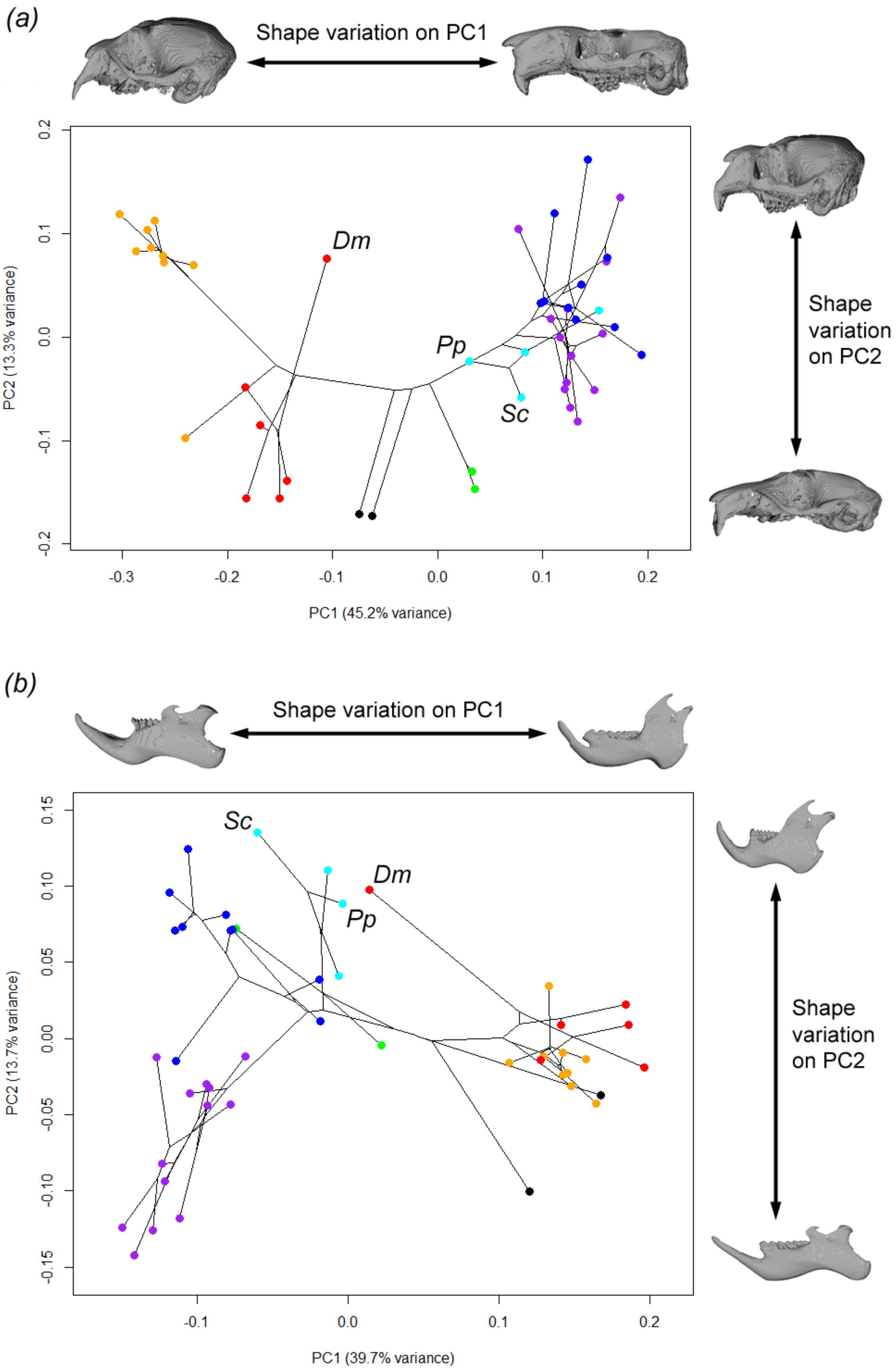
(a)
Shape variation on PC1
Shape variation on PC2
PC2 (13.3% variance)
PC1 (45.2% variance)
Dm
Pp
Sc
0.2
0.1
0.0
-0.1
-0.2
-0.3
-0.2
-0.1
0.0
0.1
0.2
(b)
Shape variation on PC1
Shape variation on PC2
PC2 (13.7% variance)
PC1 (39.7% variance)
Sc
Dm
Pp
0.15
0.10
0.05
0.00
-0.05
-0.10
-0.15
-0.1
0.0
0.1
0.2
