# Supplementary figures and images for: Convergent evolution in the Euarchontoglires
Source: Biol Lett. 2018 Aug 1;14(8):20180366. doi: 10.1098/rsbl.2018.0366 (PMC6127122; doi:10.1098/rsbl.2018.0366)

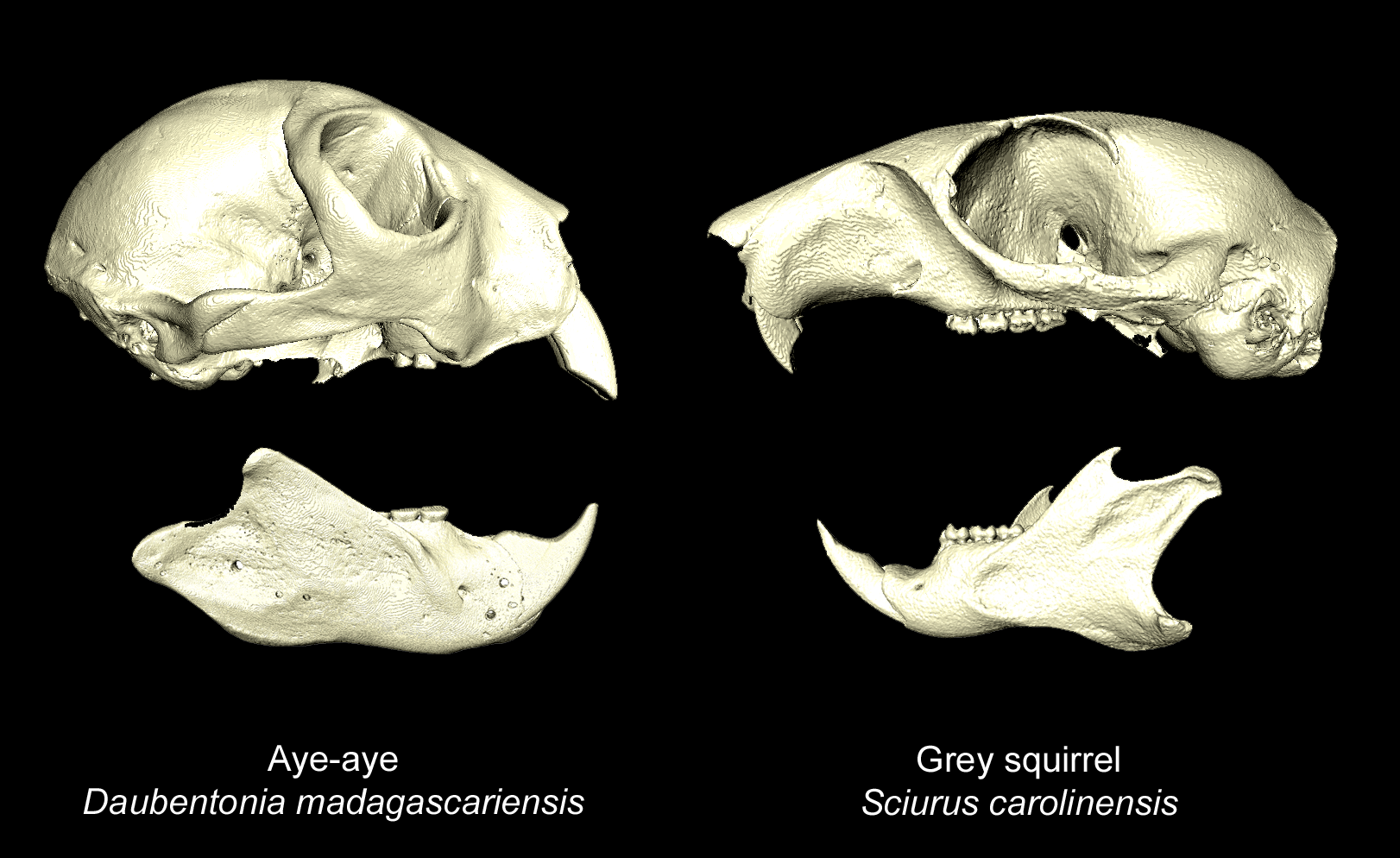

Supplement: Figure S3 [file rsbl20180366supp3.tif]
